# Research Note: Age-related effects of feeder space availability on welfare of broilers reared to 56 days of age Part 2: Blood physiological variables

**DOI:** 10.1016/j.psj.2022.101698

**Published:** 2022-01-08

**Authors:** H.A. Olanrewaju, J.L. Purswell, S.D. Collier, S.L. Branton

**Affiliations:** USDA, Agricultural Research Service, Poultry Research Unit, Mississippi State, MS 39762-5367, USA

**Keywords:** feeder-space, blood-gases, acid-base-balance, broilers, welfare

## Abstract

Consumption of poultry meat has increased dramatically due to the relative price-competitiveness as compared to other meat products. The rapid growth and increased production efficiency of modern genetic strains is perceived to negatively impact the welfare of the animal. Hematological analyses such as acid-base balance provide a thorough evaluation of the welfare in both animals and humans. This study investigated the effects of feeder space availability on welfare of broilers grown to heavy weights using blood physiological variables. The study was a randomized complete block design. In each of the 2 trials, a total of 1,440 one-d-old Ross × Ross 708 chicks (straight-run) were obtained from a commercial hatchery. Chicks were equally and randomly allocated to 32 pens based on feeder space treatment. Treatments were 4 different feeder space allocations: 2.3 (Single feeder), 2.30, 4.60, and 6.90 cm/bird. To maintain uniform bird:feeder floor space, 3 feeders were installed in each pen, except for the single feeder pen. Blood samples (3 mL) were collected from the brachial wing vein of 3 birds per pen on d 27 and 55, which were then analyzed immediately for whole blood physiological variables. The remaining blood samples were centrifuged to collect plasma that was used for corticosterone and thyroid hormones analysis. Results show there was no effect of feeder space on most of the selected physiological variables, but age had significant effects on most of the examined variables. However, all observed changes were within physiological ranges. Plasma corticosterone and blood glucose were not affected by feeder space and age, indicating absence of physiological stress. The results are in broad agreement with those reported in the literature and on homeostatic variation of broilers grown to heavy weights. In conclusion, expanding feeder allowance does not enhance the welfare of broilers grown to heavy weights.

## INTRODUCTION

Subjecting poultry to inadequate environmental conditions during poultry production has an adverse impact on production efficiency (body weight, body weight gain, feed conversion ratio), meat yield, immune response, mortality, physiological responses, and welfare (Olanrewaju et al., 2006a). As the demand for poultry and associated products increases, increasing production and production efficiencies will be critical to the continued viability of domestic poultry industries in the United States. Part of this concern centers on the question of whether feeder space elicits adaptive responses that are characteristic of physiological stress. It is important to recognize that many poultry production management strategies including feeder space availability may potentially have an adverse impact on blood homeostasis and welfare of birds. This can result in production inefficiencies, compromised animal well-being and increased animal morbidity and mortality (Seifter and Chang, 2017).

Changes in acid-base balance have been reported to signal early symptoms of diseases and influence the early manifestation of clinical signs and therapeutic effectiveness in both domestic animals and human beings (Gunnerson, 2005). The basal corticosterone and glucose levels increased in response to physiological stress have been found to be consistently and significantly higher in birds housed after infusion of adrenocorticotropic hormone (**ACTH**) and the involvement of acid-base balance in several species (Olanrewaju et al., 2006a).

We recently reported the effect of feeder space on live performance and processing yields of broiler chickens reared to 56 days of age (Purswell et al., 2021). Therefore, the objective of the present study was to investigate from the same individual birds included in our recent manuscript the effects of age-related effects of feeder space availability on selected blood physiological variables of broilers reared to 56 days of age.

## MATERIALS AND METHODS

### Bird Husbandry

All procedures relating to the use of live birds in this study were approved by the USDA-ARS Animal Care and Use Committee at the Mississippi State location. In each of the 2 trials, a total of 1,440 one-day-old Ross × Ross 708 chicks (straight-run) were obtained from a commercial hatchery. Chicks were equally and randomly allocated to 32 groups inside 32 pens based on feeder space treatment assigned as previously described by Li et al. (2021). Birds were placed onto new litter. Broilers were kept in pens from day of hatch to 56 days of age and provided a 4-phase diet on a biweekly change schedule with corn-soy diets ad libitum (NRC, 1994). Feed was continuously available while feeder space access was physically restricted with covers in some treatments which, limited the number of birds that can simultaneously feed; a full description of the experimental as previously described by Purswell et al. (2021). Lighting was provided with white 5000K LED bulbs (One-Innovation Agrishif, Plymouth, MN). Lighting intensity, photoperiod, and room temperature were adjusted as previously described (Olanrewaju et al., 2019), while minimum ventilation rates were adjusted weekly according to NPTC guidelines. Chicks were vaccinated for Marek's, Newcastle, and infectious bronchitis diseases at the hatchery. Ambient temperature was maintained at 33°C at the start of experimentation and was reduced as the birds progressed in age.

### Treatments

The 4 feeder space treatments were 2.3 cm/bird with single feeder per pen, 2.30, 4.60, and 6.90 cm/bird. To maintain similar bird:feeder space ratios across all treatments, three tube feeders equipped with typical commercial feed pans (C2Plus, Chore-Time Group, Milford, IN) were placed in each pen. Feeder space was restricted by the addition of solid plastic panels attached to the feeder grille ribs and covering the remaining grille slots with tape as previously described (Li et al., 2021). The plastic panels were constructed from ABS plastic using a 3D printer (F410, Fusion3 Design, Greensboro, NC) based upon the cross-sectional profile of the feeder pan and grille combination.

### Blood Collections and Chemical Analyses

On d 27 and 55, 3 mL blood samples were collected and analyzed as described previously (Olanrewaju et al., 2019). The mean corpuscular hemoglobin concentration (**McHc**) in grams per deciliter and arterial oxygen saturation (**SaO_2_**) were calculated as described previously (Olanrewaju et al., 2019). Plasma was collected and analyzed for corticosterone, triiodothyronine (**T_3_**), and thyroxine (**T_4_**) as described previously (Olanrewaju et al., 2019) using a universal microplate spectrophotometer (Bio-Tek Instruments Inc., Winooski, VT) with ELISA reagent assay test kits (EIA-CS Kit, Enzo Life Sciences, Farmingdale, NY) and ALPCO Diagnostics (Salem, NH) according to the manufacturer's instructions.

### Statistical Analysis

The main effects of feeder space, age and the interaction of these 2 factors if any on blood physiological variables were tested with PROC MIXED (SAS Institute Inc., 2013). Means comparisons on d 27 and 55 were assessed by least significant differences and statements of significance were based on *P ≤* 0.05 unless otherwise stated. Analyses of variance combined across days were performed to obtain treatment comparisons averaged across days and to test for treatment interactions with equal variances between days (Equation 1). The experimental unit was the treatment pen in each day.

(1) $$Y_{\text{ijk}}=\mu +\alpha_{i}+\beta_{j}+{\left(\alpha \beta \right)}_{\text{ij}}+\varepsilon_{\text{ijk}}$$

Where Y_ijk_ is the blood physiological variable, μ is the least square mean of each blood physiological variable; α_i_ is the feeder space, i = 2.3 cm/bird with single feeder per pen, 2.30, 4.60, and 6.90 cm/bird, β_j_ is the bird ages, j = d 27, d 55; (αβ)_ij_ is the interaction effect of feeder space and bird age; and ε_ijk_ is the random error. Bird age was taken as a categorical variable.

## RESULTS AND DISCUSSION

The data in this study represented the combined main effects of feeder space and age effect on major selected blood physiological variables. As shown in Table 1, there was only effect of feeder space noted on, where birds reared under 2.3 cm/bird single feeder space had higher K^+^ (*P* < 0.023) level compared to those reared under 6.90 cm/bird feeder space. In comparison with the birds raised to 27 d of old, birds reared to 55 d of age had significantly higher BW (*P* < 0.001), McHc (*P* < 0.027), Ca^2+^ (*P* < 0.024), Na^+^ (*P* < 0.001), and Cl^−^ (*P* < 0.001), along with significantly reduced pO_2_ (*P* < 0.001), SaO_2_ (*P* < 0.001), Osmo (*P* < 0.001), T_3_ (*P* < 0.001), and angap (*P* < 0.001) concentrations, which were all within physiological acid-base ranges for this species. The normal function of all physiological processes in the body depends on maintenance of appropriate acid-base balance where the body controls the abundance and distribution of acids and bases to achieve acid-base homeostasis (Bianca et al., 2021). The SaO_2_, sO_2_, and pO_2_ have direct relationships, referred to as the oxyhemoglobin (HbO_2_ or O_2_Hb) dissociation curve. The decreased in pO_2_ sO_2_, and sO_2_ observed in broilers raised to 55 days old may be due to inadequate blood oxygenation that related to their BW, which may lead to hypoxia (decreases in systemic venous pO_2_ and sO_2_). In addition, significant higher level of McHc observed in 55-day-old broilers in this study may be due to numerically increased levels of Hb and Hct. However, observed acid-base changes in the present study remained within normal acid-base homeostasis and physiological range for this species. Body fluid electrolyte concentrations, such as Na^+^, K^+^, and Cl*^−^*, and acid-base balance is interconnected and are associated with the condition producing acidosis or alkalosis in mammals, which may also be true in birds (Terzano et al., 2012). The high level of T_3_ in birds of 27 d may relate to higher feed intake for growth that support the classical role of the thyroid hormones in metabolic and physiological activities on growth and development.

**Table 1:** Combined age-related effects of feeder space on selected blood physiological variables of broilers grown to heavy weights^1^*.

	Feeder space (cm/bird)^2^						Age (days)			
Variables^3^	1	2	3	4	SEM^4^	P-value	27	55	SEM^4^	*P*-value
BW (kg)	3.872	3.924	3.871	3.927	0.129	0.797	1.245^b^	3.899^a^	0.029	0.001
pH	7.31	7.32	7.32	7.32	0.008	0.360	7.32	7.32	0.006	0.161
pCO_2_ (mm Hg)	60.04	58.34	57.58	57.00	2.507	0.992	56.16	57.22	1.773	0.676
pO_2_ (mm HG)	49.38	49.85	49.73	48.90	1.376	0.899	51.95^a^	46.98^b^	0.973	0.001
HCO_3_^−^ (mm Hg)	27.38	27.45	27.13	27.27	0.371	0.833	27.47	27.15	0.262	0.223
SaO_2_ %	72.21	72.52	72.64	73.49	1.094	0.685	74.72^a^	70.70^b^	0.774	0.001
Hb (g/dL)	8.52	8.67	8.56	8.40	0.124	0.205	8.45	8.62	0.088	0.061
Hct (%)	26.48	26.91	26.58	26.12	0.375	0.215	26.28	26.76	0.265	0.074
McHc (%)	32.11	32.21	32.12	32.17	0.027	0.271	32.16^b^	32.20^a^	0.019	0.027
GLU (mg/dL)	253.88	250.52	252.81	245.94	4.040	0.211	248.54	253.03	2.857	0.118
Osmo (mOs/kg)	313.76	313.54	314.22	314.72	1.135	0.735	315.11^a^	313.01^b^	0.803	0.001
CORT (pg/mL)	2417.5	2329.6	2286.1	2250.4	458.36	0.985	2592.6	2049.2	324.11	0.095
T_3_ (ng/mL)	2.306	2.402	2.449	2.282	0.084	0.154	2.789^a^	1.930^b^	0.059	0.001
T_4_ (mg/dL)	2.331	2.999	2.431	2.038	0.158	0.055	2.313	2.287	0.112	0.816
Ca^2+^ (mEq/L)	2.99	2.97	2.97	3.00	0.025	0.617	2.964^b^	3.01^a^	0.018	0.024
Na^+^ (mEq/L)	151.77	150.77	151.31	151.77	0.577	0.362	150.23^b^	152.45^a^	0.408	0.001
K^+^ (mEq/L)	4.60^a^	4.54^ab^	4.42^ab^	4.39^b^	0.076	0.023	4.44	4.53	0.054	0.113
Cl^−^ (mEq/L)	109.85	108.84	109.46	109.38	0.540	0.409	107.49^b^	111.32^a^	0.382	0.001
Angap (mEq/L)	19.07	18.85	19.15	19.31	0.520	0.848	19.74^a^	18.45^b^	0.368	0.001

a-b* Means with in a row not sharing a common superscript are significantly different at *P* < 0.05.

1 Table values are least squares means of eight replicate pens for each treatment.

2 Feeder space (cm/bird): 1 = 2.3 (Single feeder), 2 = 2.30, 3 = 4.60, 4 = 6.90.

3 Abbreviations: angap, anion gap; BW, Body Weight; Cl^−^, Chloride; Ca^2+^, Calcium; CORT, Corticosterone; HCO_3_^−^, Bicarbonate; Hb, hemoglobin; GLU, glucose; Hct, hematocrit; K^+^, Potassium; McHc, Mean corpuscular hemoglobin concentration; Na^+^, Sodium; Osmo, osmolality; pCO_2_, partial pressure of CO_2_; SaO_2_, saturated O_2_; T_3_, triiodothyronine; T_4_, thyroxine.

4 Pooled SEM for main effects (n = 8).

Concentrations of certain plasma hormones and metabolites such as glucose and corticosterone among others have been suggested to be sensitive indicators of stress levels in broiler chickens (Olanrewaju et al., 2006b). As shown in Table 1, no significant effect of treatments on blood glucose and plasma corticosterone concentrations in the present study suggest that these examined feeder space did not present stressors to broilers grown to heavy weights. Also, no indication of acid-base disorders (metabolic and respiratory acidosis or alkalosis) observed in the present study. Therefore, the feeder space examined in this study along with appropriate environmental management are suitable for both poultry integrators and contract growers to enhance broilers production efficiency without compromising the welfare of broilers grown to heavy weights at 56 days of age.
